# Evaluation of the levels of interleukins IL-4, IL-13, IL-5, IL-10 and IL-33 in atopic dermatitis patients with and without dupilumab therapy

**DOI:** 10.3389/fimmu.2025.1604883

**Published:** 2025-07-23

**Authors:** Jarmila Čelakovská, Eva Čermáková, Petra Boudková, Jan Krejsek

**Affiliations:** ^1^ Department of Dermatology and Venereology Faculty Hospital and Medical Faculty of Charles University, Hradec Králové, Czechia; ^2^ Department of Medical Biophysics, Medical Faculty of Charles University, Hradec Králové, Czechia; ^3^ Department of Clinical Immunology and Allergy, Faculty Hospital and Medical Faculty of Charles University, Hradec Králové, Czechia

**Keywords:** atopic dermatitis, interleukins, dupilumab, interleukin 4, interleukin 13, interleukin 10

## Abstract

**Background:**

Interleukins IL-4, Il-5, IL-10, IL-13 and IL-33 play an important role in atopic dermatitis patients. The aim of our study is to address several knowledge gaps in the understanding of interleukin dynamics in atopic dermatitis patients and the effects of dupilumab treatment.

**Method:**

We conducted an assessment of plasma levels of interleukins IL-4, IL-5, IL-10, IL-13 and IL-33 in 89 AD patients and in 44 healthy individuals as a control group. The group of AD patients consisted of 27 patients treated with dupilumab (15 men, 12 women) at a mean age of 44.8 years and 62 patients without dupilumab treatment (35 women and 27 men) at a mean age of 46.3 years. The control group consisted of 44 healthy subjects (22 men, 22 women), at a mean age of 43.3 years. Patients were treated with a standard dose of dupilumab, 300 mg s.c. every two weeks. For screening analysis of plasma levels of selected cytokines, the performance assay Human cytokine Luminex was used. Blood samples were unstimulated and stimulated with phorbol myristate acetate and ionomycin. This stimulation provides non-specific stimulation of innate and adaptive immunity cells and increases their cytokine production. The level of interleukins IL-4, IL-5, IL-10, IL-13 and IL-33 were compared in AD patients with the results in control group. Nonparametric Kruskal-Wallis analysis of variance with *post-hoc* Dunn’s test with Bonferroni modification of significance level was used.

**Results:**

The significantly higher plasma level of stimulated IL-5 was confirmed in AD patients treated with dupilumab and significantly higher plasma IL-10 levels were confirmed in both dupilumab and non-dupilumab treated patients compared to control group. Stimulated IL-4 levels are significantly higher in patients treated with dupilumab compared to patients without dupilumab. The significant difference in IL-13 and IL-33 in AD patients compared to control group was not confirmed.

**Conclusion:**

By identifying significant differences in IL-5 and IL-10 plasma levels, our study highlights potential markers that could improve AD diagnosis and treatment monitoring. Our results contribute to a deeper understanding of how dupilumab alters immune signaling and may inform the development of additional biomarkers and targeted therapies for AD.

## 1 Introduction

Atopic dermatitis (AD) is a common chronic inflammatory skin disease influenced by genetic and environmental factors, making treatment complex. The condition begins with Th2-mediated inflammation, where interleukins IL-4 and IL-13 play a crucial role. Over time, the immune response shifts from Th2 to Th1, contributing to chronic inflammation (1–4).

Early-stage AD is characterized by increased IL-4 and IL-13 production, reducing IFN-γ and TNF-α levels, which makes the skin more vulnerable to *Staphylococcus aureus* infections. *S. aureus* exacerbates inflammation through exotoxins that act as superantigens. In chronic AD, Th1 lymphocytes dominate, producing cytokines that sustain long-term inflammatory lesions (1–4). Dupilumab, a monoclonal antibody blocking IL-4Rα, helps reduce type 2 immune activity, leading to lower serum IL-4 and IL-13 levels, potentially alleviating AD symptoms (4).

In our study, we focused on IL-4, IL-5, IL-10, IL-13, and IL-33, whose roles in AD are summarized in **Table 1** (5–10). The aim is to address several knowledge gaps in the understanding of interleukin dynamics in AD and the effects of dupilumab treatment. While dupilumab blocks IL-4Rα, the expected decrease in IL-4 and IL-13 levels has not been consistently observed. Some studies even report an increase, raising questions about whether circulating levels of these cytokines truly reflect therapeutic response. For instance, Mitroi et al. observed no significant decrease in IL-4 and IL-13 serum levels, raising questions about their utility as treatment biomarkers (11). Conversely, Ariëns et al. noted an increase in IL-4 and IL-13 levels post-treatment, possibly due to elevated unbound cytokines following IL-4Rα blockade. This effect may be temporary, as prolonged suppression of IL-4Rα should eventually reduce IL-4 and IL-13 production by T cells (12).

**Table 1:** The role of interleukins IL-4, IL-5, IL-10, IL-13 and IL-33 in atopic dermatitis.

Type of interleukins	The role of interleukins in atopic dermatitis
IL-4	promotes differentiation of naive T cells into Th2 cells, stimulates B cells to produce IgE, increasing the production of specific IgE against allergens. impairs skin barrier function by reducing the expression of proteins important for skin integrity (e.g. filaggrin) (1–4).
IL-5	IL-5 plays a key role in the development, survival and proliferation of eosinophils (6). The primary producers of IL-5 are Th2 cells and ILC2, but mast cells, eosinophils, basophils, epithelial cells, and smooth muscle cells also produce IL-5 (6). IL-5 binds to a heterodimeric receptor composed of the IL-5R α subunit (IL-5Rα) and a common β subunit (βc) (6). The βc subunit is also associated with IL-3Rα and granulocyte-macrophage colony-stimulating factor (GM-CSF) Rα. In association with IL-3 and GM-CSF, IL-5 promotes proliferation, differentiation and activation of eosinophils (6).
IL-10	An anti-inflammatory cytokine that can suppress the activity of Th1 and Th2 cells and regulate the immune response to prevent excessive inflammation. It helps in controlling allergic inflammation by limiting the production of pro-inflammatory cytokines (7–10). IL-10 indirectly inhibits anti-microbial peptide expression in keratinocytes by suppressing the production of pro-inflammatory cytokines, known to induce anti-microbial peptide, by mononuclear cells.
IL-13	promotes differentiation of naive T cells into Th2 cells, contributes to IgE production and promotes mucus production and airway hyperresponsiveness. Impairs skin barrier function by reducing the expression of proteins that are essential for skin integrity (e.g. filaggrin) (1–4).
IL-33	An alarmin cytokine that can activate Th2 cells, group 2 innate lymphoid cells (ILC2s), and mast cells, leading to the production of IL-5 and IL-13. It is involved in enhancing Th2-type immune responses and promoting inflammation (5)

Interestingly, IL-22 levels correlated more strongly with clinical improvement in dupilumab-treated patients, making it a potential biomarker (12–15). While IL-4 and IL-13 share the same receptor, IL-13 can also bind to another receptor, considered mainly a decoy (16). Though blocking IL-4Rα benefits AD patients, these effects may not extend to other inflammatory conditions. A recent study suggests IL-4 and IL-13 may have anti-inflammatory effects in inflammatory arthritis (17). Additionally, dupilumab has been implicated in exacerbating myeloproliferative disorders when AD is misdiagnosed (18). Increased IL-13 levels appear associated with disease progression, while reducing IL-4 and IL-13 has been linked to improved outcomes (19).

Patient stratification based on serum biomarkers could improve therapy selection. Among 146 analyzed AD patients, only 18.5% belonged to a subgroup where IL-4 and IL-13 were predominant alongside other Th2 and Th1 markers (20). While some studies associate successful AD treatment with reduced IL-13 levels (21), Mitroi et al. reported improvements despite increased IL-13 (11). Other treatments, such as cyclosporine (22), UVA exposure (23), or tacrolimus (24), have been linked to reduced IL-13 levels. These findings suggest IL-13 inhibition could help alleviate symptoms, though further research is needed (21).

Supporting this idea, IL-13 is significantly more expressed in AD skin lesions than IL-4, though serum IL-4 and IL-13 levels may behave differently (25). Kamphuis et al. reported an initial increase in IL-4 post-dupilumab, which then gradually declined by week 16, while IL-13 levels remained largely unchanged (26).

Identifying reliable biomarkers could improve treatment monitoring and decision-making. By analyzing cytokine profiles, our study may contribute to the growing effort to classify AD patients into distinct subgroups based on immune profiles, potentially leading to personalized treatment approaches.

## 2 Materials and methods

Complete dermatological and allergological examination was performed in all patients included in the study.

All these patients were examined in the Department of Dermatology, Faculty Hospital Hradec Králové, Charles University, Czech republic.

This study was approved by Ethics committee of the Faculty Hospital Hradec Králové, Charles University of Prague, Czech Republic. Reference number is: 2021–10 P 03. The study was conducted according to the guidelines of the Declaration of Helsinki, and approved by the Institutional Review Board - Ethics committee of the Faculty Hospital Hradec Králové, Charles University of Prague, Czech Republic. Data of Approval 4 September, 2021.

### 2.1 Dermatological examination

The diagnosis of AD was determined according to Hanifin-Rajka’s diagnostic criteria. Inclusion criteria: 1) age 14 years and over 2) AD as defined by the criteria of Hanifin and Rajka. Patients with moderate and severe form of AD without dupilumab and patients with dupilumab therapy lasting at least 24 months were included. Exclusion criteria were pregnancy, breastfeeding, systemic therapy (cyclosporin, systemic corticoids, other biological therapy). The severity of AD was evaluated with Eczema. Area and Severity Index (EASI), Scoring of atopic dermatitis (SCORAD), the subjective assessment of eczema by the patient was also processed with POEM index (Patient Oriented Eczema Measure) and with DLQI index (Dermatology Life Quality Index) (27).

#### 2.1.1 Patients

In our study, interleukins IL-4, IL-5, IL-10, IL-13 and IL-33 were examined in 89 AD patients. (42 men and 47 women) with a mean age of 46.3 years. This group consisted of 27 patients treated with dupilumab (15 men, 12 women) at a mean age of 44.8 years and 62 patients without dupilumab treatment (35 women and 27 men) at a mean age of 46.3 years.

At the same time, 44 healthy subjects were examined with this study group, (22 men, 22 women), age 43.3 years s.d. 9.5 years. Complete dermatological and allergolocical examination was performed in all patients included in the study.

In patients without dupilumab, the AD severity and quality of life were assessed every 3 months for 1 year. In dupilumab-treated patients, AD severity and quality of life were assessed every 3 months during the 1 year prior to dupilumab treatment and every 3 months during the 24 months of dupilumab treatment. Patients were treated with a standard dose of dupilumab, i.e. 300 mg s.c. every two weeks. We included patients who had been treated with dupilumab for at least 18 months; only the clinical responders were included in the study.

The characteristic of patients with mean SCORAD, EASI, POEM and DLQI values are shown in **Table 2**.

**Table 2:** Characteristic of atopic dermatitis patients.

Patient characteristics	Dupilumab untreated patients	Dupilumab treated patients	
**Age**	**mean age of 46.3 years** (24.2- 52.3)	mean age of 44.8 years (31.6-48.3)	
**Number of patients**	62 (27 men, 35 women)	27 (15 men, 12 women)	
**SCORAD**	**32.5** (26.5-38.7)	**34.1** (30.5-45.2)	Before dupilumab therapy
		**9.5** (7.1-18.2)	Average value after 1.5 years of treatment with dupilumab
**EASI**	**30.3** (26.8-38.5)	**33.1** (30.1-44.2)	Before dupilumab therapy
		**9.1** (8.2-17.2)	Average value after 1.5 years of treatment with dupilumab
**POEM**	**13.9** **(10**–18)	**14.3** (12–21)	Before dupilumab therapy
		**4.1** (2 -6)	Average value after 1.5 years of treatment with dupilumab
**DLQI**	**12.9** (9 – 16)	**15.5** (11-20)	Before dupilumab therapy
		**3.4** (1-5)	Average value after 1.5 years of treatment with dupilumab
**Previous systemic treatment**	62 patients (100%) - antihistamines - no other previous systematic treatment	27 patients (100%) - antihistamines - cyclosporin	
**Previous local treatment**	local corticosteroid therapy with antiseptics, emollients, topical immunomodulators	local corticosteroid therapy with antiseptics, emollients, topical immunomodulators	
**Asthma bronchiale**	26 patients (41.9%)	15 patients (55.5%)	
**Allergic rhinitis**	42 patients (67.7%)	17 patients (62.9%)	
Control group - 44 healthy subjects (22 men, 22 women), age 43.3 years s.d. 9.5 years			

The average values (minimal, maximal values) of SCORAD, EASI, POEM, DLQI are recorded. SCORAD, Scoring of atopic dermatitis; EASI, Eczema Area and Severity Index; POEM, Patient Oriented Eczema Measure; DLQI, Dermatology Life Quality Index. The mean values are recorded in bold.

#### 2.1.2 Control group

As a control group, we examined- 44 healthy subjects (22 men, 22 women), age 43.3 years s.d. 9.5 years, blood donors at Faculty Hospital Hradec Králové, Charles University, Czech republic. Total IgE was examined in control group and only healthy subjects with negative total IgE were included.

### 2.2 The laboratory examination

For screening analysis of selected cytokines, the performance assay Human cytokine Luminex^®^: IL-4, IL-5, IL-10, IL-13, IL-33 (R&D systems, Minneapolis, MN, USA) was used. Blood samples were unstimulated and stimulated with PMA (phorbol myristate acetate) and IO (ionomycin). This stimulation provides non-specific stimulation of innate and adaptive immunity cells and increases their cytokine production. Dosage and *in vitro* stimulation with PMA and IO were performed based on previous historical optimization of the method and knowledge from the professional literature (29).

Peripheral blood was collected from patients into a tube with the anticoagulant Heparin. Subsequently, the blood was added to the X-VIVO culture medium in a ratio of 1:1. Two culture tubes were used for each patient. The first tube was always stimulated by adding 25 µl PMA (concentration 0.25 µg/ml) and 20 µl IO (concentration 2 µg/ml), the second tube was left without stimulation. Both tubes were incubated at 37°C for 4 hours. Subsequently, the samples were centrifuged to obtain stimulated and unstimulated plasma and frozen. After collecting a certain number of patients, the plasma samples were thawed and used for Luminex tests.

Cytokine levels were determined using xMap technology with a Bio-Plex 200 system (Bio-Rad, Hercules, CA, USA), analysis of the results was performed using xPONENT^®^ 4.2. analysis software (Luminex Corporation, Austin, TX, USA) (28). The Luminex test was used as a multiplex test. The cut-off value for valid values of individual cytokines was determined according to the values of the standard curves for individual interleukins. The number of subjects for detectable levels of individual cytokines differed within individual interleukins and also within unstimulated vs. stimulated samples. Within stimulated samples, of course, the proportion of subjects with detectable levels was higher than the proportion of subjects in unstimulated samples. It is necessary to take into account that the activation of immune cells for the production of individual cytokines may differ in patients in the *in vivo* mode and in the artificially induced stimulus *in vitro*. This is therefore only an expected reaction of immune cells.

According to the strictly followed instructions for the Luminex test, the standard samples were prepared using three-fold dilutions. The standard curve therefore has a rather exponential character. Example of a standard curve for IL-4 is recorded in **Supplementary Figure 1**.

### 2.3 Allergological examination

To evaluate the presence of other atopic diseases, we performed a comprehensive allergological examination.

#### 2.3.1 Bronchial asthma

The diagnosis of bronchial asthma (AB) was made according to the guidelines of the Global Initiative for Asthma (GINA) at the allergy outpatient clinic of the Institute of Clinical Immunology and Allergy of the University Hospital Hradec Králové. (Global Strategy for Asthma Treatment and Prevention - 2015 Update. www.ginasthma.com).

#### 2.3.2 Allergic rhinitis

The evaluation of allergic rhinitis (AR) was performed according to allergy examination and personal history at the Department of Clinical Immunology and Allergy, University Hospital Hradec Kralove, Czech Republic. AR was defined as a process involving three cardinal symptoms: sneezing, nasal obstruction and mucus discharge. Symptoms appear when an allergic patient is exposed to an allergen.

#### 2.3.3 Examination of specific IgE to allergens and molecular components

To determine whether there is a difference in sensitization to allergens in patients treated and untreated with dupilumab, we examined specific IgE antibodies. We analyzed the results of specific IgE to molecular components of food and inhalant allergens in all AD patients (with and without dupilumab therapy) included in the study. The specific IgE was examined with the use of ALEX2 Allergy Xplorer test.

### 2.4 Statistical analysis

The laboratory results (the level of interleukins IL-4, IL-5, IL-10, IL-13 and IL-33) were compared in AD patients (treated or not treated with dupilumab) with the results in control group. The hypothesis of agreement was tested against the alternative that at least two groups differ from each other. In this case, this means that the medians in all three groups match, the alternative hypothesis is that the medians of at least two groups differ. Nonparametric Kruskal-Wallis analysis of variance with *post-hoc* Dunn’s test with Bonferroni modification of significance level was used. The respective significance levels are highlighted.

To assess the difference in the number of positive specific IgE results between patients with and without biological treatment. We used Pearson´s Chi- Square test and Fisher´ s exact test.

We used statistical software: NCSS 2023 Statistical Software (2023). NCSS, LLC. Kaysville, Utah, USA, ncss.com/software/ncss. The level of significance was α=0.05.

## 3 Results

### 3.1 Characteristic of patients and control group

The characteristic of AD patients (including evaluating the severity of AD and quality of life, the number of patients with bronchial asthma and allergic rhinitis) is recorded in **Table 2**. In **Supplementary Table 1** we show number of patients with positive results of specific IgE to allergens and molecular components. The difference in the number of patients with positive results of specific IgE in AD patients with and without dupilumab therapy was not confirmed.

The representation of AD patients (treated or non-treated with dupilumab) did not differ in terms of age, gender and onset of AD. Likewise, the representation of the control group did not differ in terms of age and gender. AD severity did not differ between the two groups of patients with AD before starting dupilumab therapy.

Patients treated with dupilumab had moderate and severe AD before starting biologic therapy; after starting dupilumab, skin findings improved significantly (**Table 2**). In addition to dupilumab, which is applied 300 mg s.c. every two weeks, they are treated with topical therapy to hydrate the skin. Patients without dupilumab therapy are treated with emollients, topical corticosteroids with antiseptics for acute exacerbations.

### 3.2 The level of interleukins IL-4, IL-5, IL-10, IL-13 and IL-33 in AD patients – results of statistical analysis

The difference in IL-13 and IL-33 in AD patients compared to control group was not confirmed. We demonstrated significant differences in plasma levels of interleukins between AD patients and the control group only for interleukins 4, 5 and 10. The graphs demonstrate the significant results of these interleukins. Because data are nonnormal distributed, we show the median, the box is the 25th and 75th percentile.

**Figure 1:**
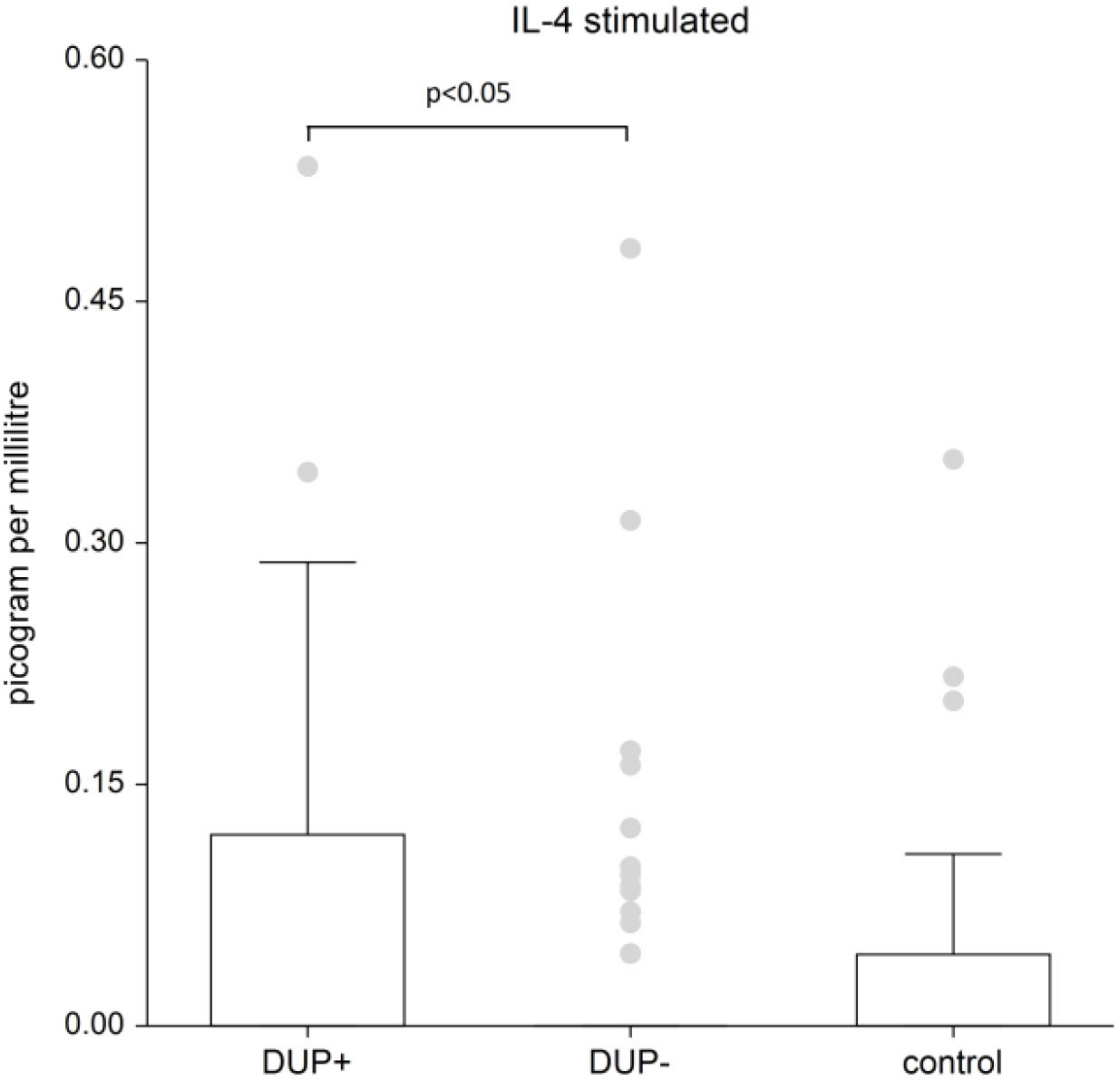
The level of IL- 4. Shows stimulated plasma samples of IL-4. IL-4 levels are significantly higher in patients treated with dupilumab compared to AD patients without dupilumab (p<0.05); when comparing IL-4 levels to the control group, there wasn’t found significant difference in patients without dupilumab; in patients with dupilumab the difference wasn’t found statistically significant also, but the p-value is on borderline of significance (p-value=0.0584). Box whisker plot of median - the top edge of the box is the 75th percentile, median=0, dots are outliers.

**Figure 1** shows the plasma level of stimulated IL-4. IL-4 levels are significantly higher in patients treated with dupilumab compared to AD patients without dupilumab (p<0.05); when comparing IL-4 levels to the control group, there wasn’t found significant difference in patients without dupilumab; in patients with dupilumab the difference wasn’t found statistically significant also, but the p-value is on borderline of significance (p-value=0.0584).

**Figure 2:**
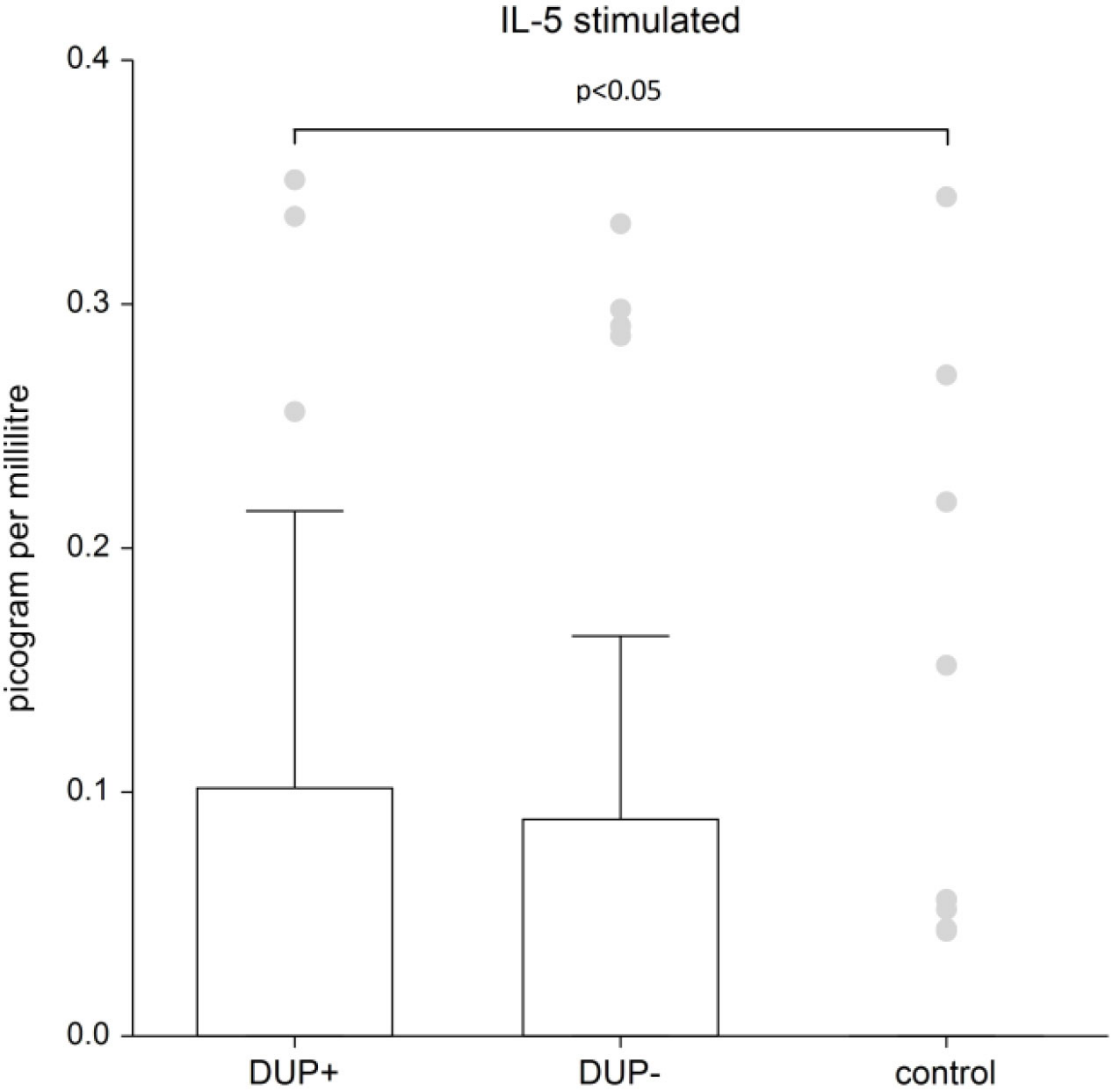
The level of IL- 5. Shows stimulated plasma samples of IL-5. IL-5 levels are significantly higher in AD patients with dupilumab compared to control group (p=0.0245). There is no significant difference between AD patients without dupilumab to control group. Box whisker plot of median - the top edge of the box is the 75th percentile, median=0, dots are outliers.

**Figure 2** shows the plasma level of stimulated IL-5. IL-5 levels are significantly higher in AD patients with dupilumab compared to control group (p=0.0245). There is no significant difference between AD patients without dupilumab to control group.

**Figure 3:**
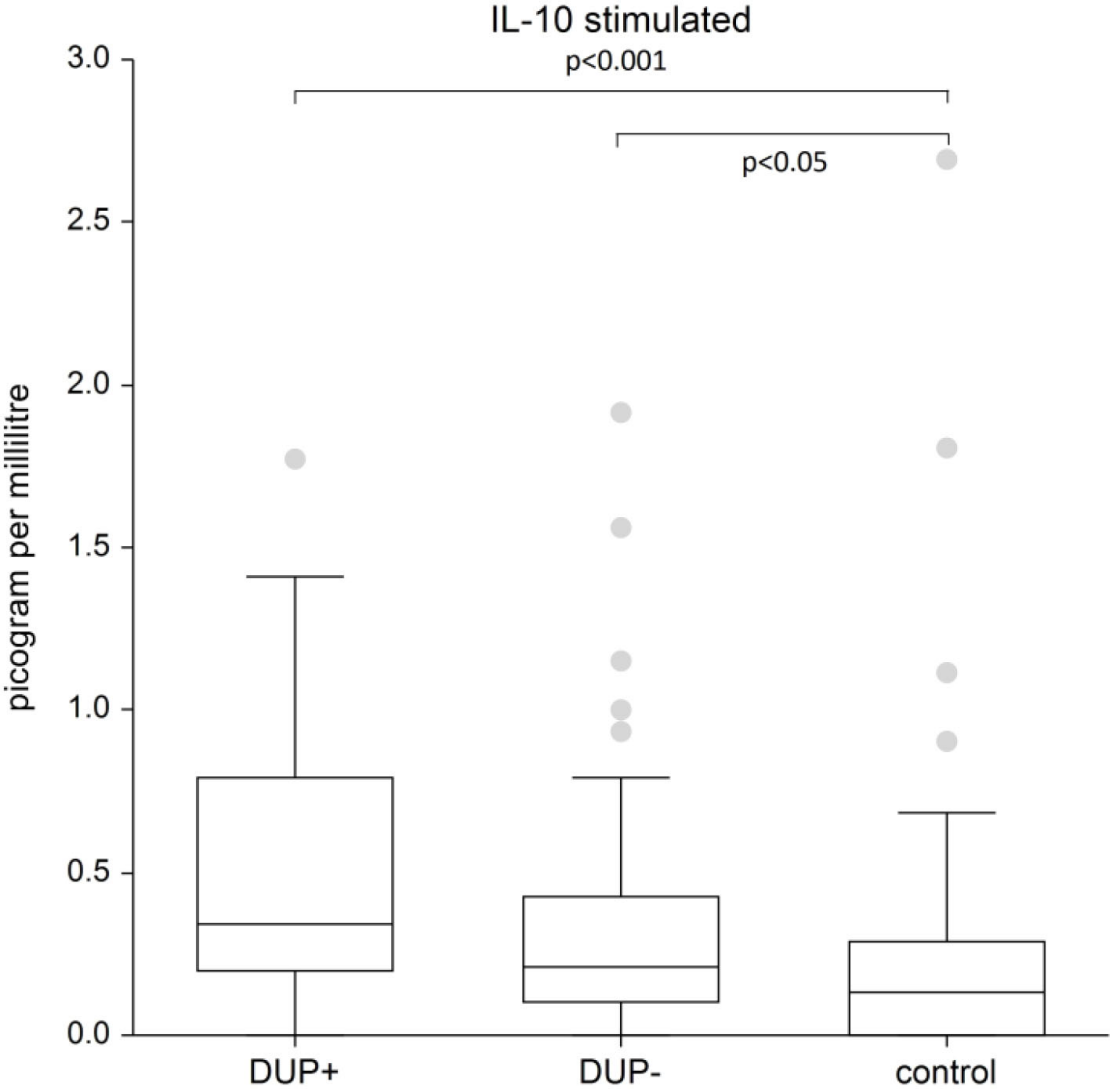
The level of IL-10. Shows stimulated plasma samples of IL-10. We recorded a significantly higher plasma level of IL-10 in AD patients with dupilumab compared to control group (p<0.001) and in AD patients without dupilumab compared to control group (p<0.05). The box is the 25th and 75th percentile, the line in the box is the median (50th percentile), dots are outliers,.

**Figure 3** shows the plasma level of stimulated IL-10. We recorded a significantly higher plasma level of IL-10 in AD patients with dupilumab compared to control group (p<0.001) and in AD patients without dupilumab compared to control group (p<0.05).

As an explanation for the graphs, we add **Table 3** and the **Supplementary Table 2**, where we show the measured interleukin values ​​for both stimulated and unstimulated samples.

**Table 3:** Basic statistical characteristics for interleukins (in picogram per milliliter) in patients with and without dupilumab therapy.

Interleukins	Dupilumab yes 27 patients			Dupilumab no 62 patients			Control group 44 healthy subjects		
	percentile			percentile			percentile		
	50th	25th	75th	50th	25th	75th	50th	25th	75th
IL 4 uns.	0	0	0	0	0	0	0	0	0
IL-4 s	0	0	0.119	0	0	0	0	0	0.0442
IL 13 uns.	0	0	0	0	0	0	0	0	0
IL 13 s	0	0	5.83E-16	0	0	0	0	0	0
IL 5 uns	0	0	0	0	0	0	0	0	0
IL 5 s	0	0	0.102	0	0	0.089	0	0	0
IL 10 uns.	0	0	0	0	0	0	0	0	0
Il 10 s	0.342	0.196	0.79	0.208	0.1	0.427	0.132	0	0.286
IL 33 uns.	0	0	0	0	0	0	0	0	0
IL 33 s	0	0	0	0	0	0	0	0	0

Given the rejected normality, we report the median (50th percentile) and the interquartile range (25th, 75th percentile) as a measure of variability.

of interleukins IL-4, IL-5, IL-10, IL-13 and IL-33 in AD patients (with and without dupilumab therapy). Given the rejected normality, we report the median and the interquartile range as a measure of variability in **Table 3**. Since the median is equal to zero in the vast majority of cases, we added the mean and standard deviation in **Supplementary Table 2**. We show the statistical comparison of interleukins between AD patients with and without biological therapy in comparison to control group.

The levels of IL-5 unstimulated and IL-10 unstimulated is zero in all groups, so comparison could not be made.

## 4 Discussion

The purpose of our study is to evaluate the interleukin levels in AD patients. Patients both with and without dupilumab treatment were included in the study. We included patients who had been treated with dupilumab for at least 2 years. All of these patients had moderate to severe AD before starting dupilumab therapy. Regarding the severity of AD, the skin findings are significantly improved in patients with dupilumab and these patients have a mild form of AD, whereas patients without dupilumab treatment suffer from a moderate to severe form of AD.

We focused on the following interleukins IL-4, IL-5, IL-10, IL-13 and IL-33. We expected mainly differences in IL-4 and IL-13 levels, but surprisingly we confirmed the differences in IL-5, IL-10 and borderline in IL-4 levels. The significantly higher level of IL-5 was confirmed in AD patients treated with dupilumab compared to control group. Significantly higher IL-10 levels were confirmed in both dupilumab and non-dupilumab patients; interestingly, IL-10 levels were highest in dupilumab-treated patients, The higher level of IL- 4 stimulated in patients treated with dupilumab is on borderline of statistical significance compared to the control group (p-value 0.05839); moreover, IL-4 levels are significantly higher in patients treated with dupilumab compared to patients without dupilumab (p<0.05).

Most research on dupilumab has focused on its role in inhibiting IL-4 and IL-13, which are key factors in the inflammatory response in AD (30). Dupilumab significantly reduces levels of biomarkers such as thymus-regulated chemokine and activation (TARC), eotaxin-3, total immunoglobulin E (IgE), and periostin (31). These reductions occur in multiple conditions, including AD, asthma, chronic rhinosinusitis with nasal polyps (CRSwNP), and eosinophilic esophagitis (EoE). The effect on blood eosinophil levels varies by disease. In AD, the changes are minimal, whereas in asthma and in CRSwNP there is a transient increase followed by a decrease to sub-baseline values. A significant decrease in eosinophil levels is observed in EoE (32). Dupilumab helps to restore the skin barrier by reducing the production of certain chemokines and cytokines, leading to fewer inflammatory cells infiltrating the skin (33). Although the primary targets are IL-4 and IL-13, inhibition of dupilumab may indirectly affect other cytokines and immune responses, potentially reducing overall inflammation and improving clinical outcomes (32).

Our study provides several unique insights compared to previous research on atopic dermatitis and interleukin dynamics: Our study clarifies dupilumab’s impact on cytokines. While prior studies assumed IL-4 and IL-13 levels would consistently decrease with dupilumab treatment (11 – 26), our study reveals a more nuanced cytokine response—particularly the unexpected increase in IL-4 and IL-5 in stimulated samples. Unlike many studies that focus solely on IL-4 and IL-13, our research also examines IL-5, IL-10, and IL-33, providing a more comprehensive picture of immune regulation in AD. The study’s inclusion of a healthy control group allows for a direct comparison of cytokine fluctuations, helping to distinguish AD-specific immune dysregulation from baseline immune activity. By identifying significant differences in IL-5 and IL-10 plasma levels, our study highlights potential markers that could improve AD diagnosis and treatment monitoring. Our results contribute to a deeper understanding of how dupilumab alters immune signaling and may inform the development of additional biomarkers and targeted therapies for AD.

Our study confirmed higher plasmatic IL-10 levels in both dupilumab-treated and untreated atopic dermatitis patients compared to the control group. Notably, there are no prior studies directly linking dupilumab to increased IL-10 in AD patients. IL-10 is a pleiotropic cytokine essential for immune modulation, primarily acting as an anti-inflammatory mediator via the Jak1/Tyk2-STAT3 pathway, but it can also have immunostimulatory effects. Given its role in hyperinflammatory conditions, IL-10 could be significant in diseases like cancer, COVID-19, and post-COVID-19 syndrome (34, 35). IL-10 has been shown to exert a wide range of effects on target cells including a reduction in the synthesis of AMPs (9, 10, 34). AMPs are important components of cutaneous innate immunity that exhibit antimicrobial and immunomodulatory activities that play an important role in the pathogenesis of AD. Recent studies have shown that dysregulation of AMPs could be one of the factors contributing to the development of AD, leading to the increased susceptibility to skin infections and disease exacerbation (9, 10, 35). AMPs including defensins, cathelicidin, psoriasin, dermcidin, and ribonucleases, play a crucial role in skin immunity but may be dysregulated in atopic dermatitis. Their antimicrobial and immunomodulatory effects make them promising for predicting disease severity and developing new treatments. IL-10, initially defined as an inhibitor of cytokine synthesis, is now recognized as a key immunoregulatory cytokine with both anti-inflammatory and immunostimulatory properties. Its effects depend on target cells, timing, and secretion site, influencing immune responses through multiple signaling pathways. Due to its dual nature, any therapeutic approach targeting IL-10 must be carefully balanced to avoid unintended immune activation or suppression (36–38).

In our study, the increase in IL-10 levels in dupilumab-treated patients may be a compensatory mechanism of the immune system that offsets reduced pro-inflammatory IL-4 and IL-13 signals. This helps to further suppress inflammation and promote a more controlled immune response, contributing to the therapeutic effects of dupilumab in AD patients. Another finding in our study is the significantly higher level. of IL-5 in AD patients treated with dupilumab compared to control group. On the other hand, plasma IL-5 levels in patients not treated with dupilumab did not differ from the control group. IL-5 is primarily responsible for the growth and survival of eosinophils. In AD, eosinophils are part of the inflammatory process, and their numbers are often elevated. Dupilumab does not directly target IL-5 or its receptor, so while it dampens the effects of IL- 4 and IL-13, it may not reduce the activity of IL-5. This could lead to a relative increase in IL-5 levels as dupilumab selectively suppresses other parts of the Th2 response. Another explanation may be, that patients with more severe AD may have higher baseline levels of IL-5. When treated with dupilumab, their IL-5 levels might remain elevated compared to untreated patients and healthy controls, even if there is a clinical improvement in their condition (39). Studies evaluating plasma IL-5 levels in AD patients treated with dupilumab have been performed (40). This study found that dupilumab significantly reduced IL-5 levels, reinforcing its Th2 inflammatory pathway inhibition. It also lowered all examined biomarkers and improved the transcriptome of AD lesions (40).

Although the higher IL- 4 stimulated levels in patients treated with dupilumab in our study are not statistically significant compared to control group; the p-value is on borderline of statistical significance. On the other hand, studies have shown that patients treated with dupilumab show a significant reduction in IL-4 levels compared with those not treated with the drug. This reduction is associated with improved clinical outcomes such as reduced eczema severity, reduced pruritus and overall better skin condition (41, 42). However, in our study the presence of higher IL-4 levels in patients treated with dupilumab compared to those not treated with it and to control group might seem counterintuitive. The body might increase the production of IL-4 as a compensatory response to the blockade of IL-4 signaling. This is a common phenomenon where the body attempts to maintain homeostasis by producing more of a blocked substance. In our previous study with the same group of patients we confirmed in AD patients treated with dupilumab the higher count of CD16^+^ eosinophils (activated eosinophils) and the higher association between the count of eosinophils (absolute and relative) and the expression of CD23 marker on B cells. It suggests that IL-4 production by eosinophils may play a role in B lymphocyte activation (43). Our results from this previous study suggest that eosinophils may be to maintain IL-4 production, because this also occurs through eosinophils. Dupilumab blocks the production of IL-4, which is important for the activation of B lymphocytes. To maintain B cell activation with IL-4, we can hypothesize that this occurs through IL-4 production by eosinophils, while the IL-4 produced by basophils could be blocked by dupilumab. This corresponds to an increased relative count of CD16^+^ eosinophils (30.0%) in patients with AD with dupilumab (43). The results of our current study are consistent with our expectation and hypothesis that IL-4 levels are elevated in patients treated with dupilumab.

An analysis comparing 11 studies shows that treatment with dupilumab resulted in a transient increase in mean blood eosinophil counts in patients with asthma or AD, which usually decreased to baseline or below over time and was generally not associated with clinical symptoms or an effect on efficacy (44) As IL-4 and IL-13 do not mediate eosinophil maturation and release into the blood, this reduction in eosinophil migration into the tissue may lead to a transient increase in blood eosinophil counts (44).

According to the literature, eosinophils produce significant amounts of IL-4 (45). Piehler et al. demonstrated an immunoregulatory role of eosinophils that contribute to IL-4 dependent immunopathological features during murine pulmonary *C. neoformans* infection, and provided evidence for previously unrecognized features of eosinophils during bronchopulmonary infection (45).

In general another explanation for higher level of IL-4 may be the fact, that if IL-4 levels are measured shortly after starting dupilumab treatment, there might be a temporary increase before the levels stabilize. We included in our study AD patients who had been treated with dupilumab for at least 2 years; we believe this is long enough for IL- 4 levels to decline. Dupilumab blocks the IL-4 receptor, not the production of IL-4 itself. Therefore, IL-4 can still be produced, but because of the blocked receptor it cannot act, leading to an accumulation of IL-4 in the bloodstream. However, the accumulation of IL-4 in the bloodstream is usually not a problem. If IL-4 cannot bind to its receptor, its production may be reduced due to feedback mechanisms in the immune system (46). Another study confirmed, that patients with moderate-to-severe AD treated with dupilumab significantly reduced the expression of genes involved in type 2 inflammation (IL-13, IL-31, CCL17, CCL18, and CCL26), and Th17/Th22 activity and increased expression of lipid metabolism and barrier genes (ELOVL3, loricrin (LOR), claudins, filaggrin (FLG) (47).

As for IL-13, cytokine with several important roles in the immune system, in our study the level is highest in patients with dupilumab (0.128) compared to patients without dupilumab (0.097) and to the control group (0.03), but the difference is not statistically significant. The available clinical data and studies on IL-4 and IL-13 serum levels during dupilumab treatment are limited. Furthermore, the existing data tend to be contradictory. Mitroi et all. tried to follow the dynamics of IL-4 and IL-13 and possibly identify them as potential reliable biomarkers in AD (11). IL-4 and IL-13 share the same receptor, although IL-13 may also bind to a different receptor, which is thought to be primarily a decoy receptor (16). Mitroi et all. have shown in the their study, that inhibition of the receptor for IL-4 and IL-13 leads to an increase in serum concentrations of both ILs. The explanation for this phenomenon is based on a recently published article by Melo-Cardenas et al, in which the authors show that progression of myeloproliferative disorders is associated with increased IL-13 levels, whereas reductions in IL-4 and IL-13 are associated with reduced features of these disorders (19). In addition, interleukin 13 is involved in gastrointestinal diseases such as ulcerative colitis and eosinophilic esophagitis (48). It may influence intestinal inflammation and the immune response to intestinal pathogens and is involved in the process of fibrosis, which is the hickening and scarring of connective tissue. This can occur in various organs, including the lungs, where IL-13 contributes to conditions such as idiopathic pulmonary fibrosis.

IL-13 also has protective effects in the nervous system, which may aid in the development of the nervous system (49).

IL-33 is a new member of the IL-1 superfamily of cytokines that is expressed by mainly stromal cells, such as epithelial and endothelial cells, and its expression is upregulated following pro-inflammatory stimulation (50). In our study the level of IL-33 (in picogram per milliliter) is highest in patients with dupilumab (0.10) compared to patients without dupilumab (0.02) and to the control group (0.00), but the difference is not statistically significant. It functions as an alarm signal (alarmin) that is released when cells or tissue are damaged to alert immune cells expressing the ST2 receptor (IL-1RL1). The main targets of IL-33 *in vivo* are tissue immune cells such as mast cells, innate lymphoid group 2 cells (ILC2) and regulatory T cells (Treg). Other cellular targets are T helper 2 (Th2) cells, eosinophils, basophils, dendritic cells, Th1 cells, CD8+ T cells, NK cells, iNKT cells, B cells, neutrophils and macrophages. IL-33 thus emerges as a key immune modulator with pleiotropic activities in type 2, type 1 and regulatory immune responses and with important roles in allergic, fibrotic, infectious and chronic inflammatory diseases (50, 51). Thus, the effects of IL-33 are either pro-inflammatory or anti-inflammatory depending on the disease and model.

In our study, the difference in the level of IL-33 is not statistically significant in AD patients compared to control group. In AD patients, IL-33 might be part of a compensatory mechanism that maintains its levels despite the suppression of other inflammatory pathways. In dupilumab treated patients, IL-33 may be regulated by different pathways that are not directly affected by IL-4 or IL-13 blockade. This means that while dupilumab effectively reduces inflammation mediated by IL-4 and IL-13, it does not impact the pathways controlling IL-33 production.

### 4.1 Limitations

1) Given the differing sample sizes- 27 treated AD patients with dupilumab, 62 untreated patients, and 44 controls - we evaluated the effect of size among the patients groups The difference in the frequency of the groups had the effect of reducing the overall power of the test, i.e. there were more false negative results (failure to reject the hypothesis of agreement) than would have been the case with evenly distributed frequencies. The control group makes up about a third of the set, which corresponds to the optimum, unfortunately in the sick group the patients are not evenly divided into groups.

2) A limitation of the study is that we do not compare interleukin levels in the same group of patients (before and after two years of dupilumab therapy). However, we believe that the two groups of patients are comparable (in terms of the presence of other atopic diseases, they come from the same region, sensitization to allergens). Other limitation is the cross-sectional design. Cross-sectional studies are observational studies that analyze data from a population at a single point in time. They are useful for obtaining preliminary evidence when planning a future advanced study (52). Our study provides information about the levels of interleukins in AD patients (with and without dupilumab therapy); this information will be useful for planning a cohort study.

## 5 Conclusion

The higher level of stimulated IL-5 was confirmed in AD patients treated with dupilumab and higher IL-10 levels were confirmed in both dupilumab and non-dupilumab treated patients compared to control group. Stimulated IL-4 levels are significantly higher in patients treated with dupilumab compared to patients without dupilumab. When comparing IL-4 levels to the control group, there wasn’t found significant difference in patients without dupilumab; in patients with dupilumab the difference wasn’t found statistically significant, but the p-value is on borderline of significance. The difference in IL-13 and IL-33 in AD patients compared to control group was not confirmed.

By identifying significant differences in IL-5 and IL-10 plasma levels, our study highlights potential markers that could improve AD diagnosis and treatment monitoring. Our results contribute to a deeper understanding of how dupilumab alters immune signaling and may inform the development of additional biomarkers and targeted therapies for AD.

## Data Availability

The raw data supporting the conclusions of this article will be made available by the authors, without undue reservation.

## Supplementary material

Supplementary Table 1 Characteristic of patients– number of patients with positive results of specific IgE to allergens and molecular components. The difference in the number of patients with positive results of specific IgE in AD patients with and without dupilumab therapy was not confirmed. Aln g 1 (PR-10 protein, alder), Bet v 1 (PR-10 protein, birch), Cor a 1.0401 (PR-10 protein, hazel pollen), Phl p 1 (Beta expansin, Timothy grass), Lol p 1 (Beta expansin, rye grass), Sec c_pollen (cultivated rye, pollen), Can f 1 (Lipocalin, dog), Der p 2 (NPC2 family, house dust mite), Der f 2 (NPC2 family, house dust mite), Der p 23 (peritrophin-like protein domain, house dust mite), Fel d 1 (uteroglobin, cat), Alt a 1 (unknown, Alternaria alternata), Gal d white – egg, Gal d yolk – egg, Mala s 11 (Mn superoxide dismutase, Malassezia sympodialis).

Supplementary Table 2 The statistical comparison of the levels of interleukins between patients with and without dupilumabl therapy compared to control group. We show the mean and standard deviation (s.d.) of interleukins (in picogram per milliliter). The levels of IL5 unstimulated and IL10 unstimulated is zero in all groups, so comparison could not be made. The hypothesis of agreement was tested against the alternative that at least two groups differ from each other. Nonparametric Kruskal-Wallis analysis of variance with *post-hoc* Dunn’s test with Bonferroni modification of significance level was used. The respective significance levels are highlighted. We show a statistically significant difference when comparing the groups: 1) AD patients with dupilumab versus AD patients without dupilumab (DUP yes/DUP no). 2) AD patients without dupilumab versus control (DUP no/control). 3) AD patients with dupilumab versus control (DUP yes/control. Explanation: uns. - unstimulated interleukins, s.- stimulated interleukins, KW test = Kruskal-Wallis analysis. DUP: dupilumab.

Supplementary Figure 1 According to the strictly followed instructions for the Luminex test, the standard samples were prepared using three-fold dilutions. The standard curve therefore has a rather exponential character. Example of a standard curve for IL-4 is recorded in Complement material to section 2.2. We also attach an example of specifically stated concentration values:
