# Supplementary material for: Evaluation of the levels of interleukins IL-4, IL-13, IL-5, IL-10 and IL-33 in atopic dermatitis patients with and without dupilumab therapy
Source: Front Immunol. 2025 Jul 23;16:1604883. doi: 10.3389/fimmu.2025.1604883 (PMC12325203; doi:10.3389/fimmu.2025.1604883)

**Complement material to section 2.2.**

According to the strictly followed instructions for the Luminex test, the standard samples were prepared using three-fold dilutions. The standard curve therefore has a rather exponential character. Example of a standard curve for IL-4 is recorded in Complement materiál to section 2.2.


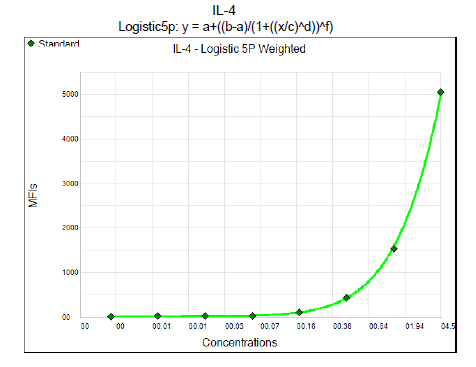


We also attach an example of specifically stated concentration values:


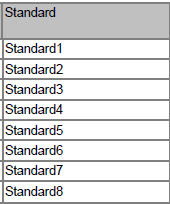

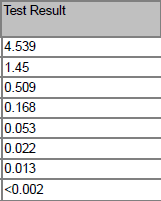

Supplement: Supplementary Table 1 — Characteristic of patients– number of patients with positive results of specific IgE to allergens and molecular components. The difference in the number of patients with positive results of specific IgE in AD patients with and without dupilumab therapy was not confirmed. Aln g 1 (PR-10 protein, alder), Bet v 1 (PR-10 protein, birch), Cor a 1.0401 (PR-10 protein, hazel pollen), Phl p 1 (Beta expansin, Timothy grass), Lol p 1 (Beta expansin, rye grass), Sec c_pollen (cultivated rye, pollen), Can f 1 (Lipocalin, dog), Der p 2 (NPC2 family, house dust mite), Der f 2 (NPC2 family, house dust mite), Der p 23 (peritrophin-like protein domain, house dust mite), Fel d 1 (uteroglobin, cat), Alt a 1 (unknown, Alternaria alternata), Gal d white – egg, Gal d yolk – egg, Mala s 11 (Mn superoxide dismutase, Malassezia sympodialis). [file DataSheet1.docx]
