# Supplementary material for: Evaluation of the levels of interleukins IL-4, IL-13, IL-5, IL-10 and IL-33 in atopic dermatitis patients with and without dupilumab therapy
Source: Front Immunol. 2025 Jul 23;16:1604883. doi: 10.3389/fimmu.2025.1604883 (PMC12325203; doi:10.3389/fimmu.2025.1604883)

**Complement to Table 2.** Characteristic of patients– number of patients with positive results of specific IgE to allergens and molecular components. The difference in the number of patients with positive results of specific IgE in AD patients with and without dupilumab therapy was not confirmed.

| **Molecular allergens** | **Number of patients with positive results of specific IgE to molecular allergens** | | **Fisher´s exact test**  **Prob level** |
| --- | --- | --- | --- |
|  | **AD patients without dupilumab**  (62 patients) | **AD patients with dupilumab**  (27 patients) | *p-value* |
| **Aln g 1** | 28 (45.1 %) | 12 (44.4 %) | *1.000* |
| **Bet v 1** | 28 (45.1 %) | 11 (40.7 %) | *1.000* |
| **Cor a 1.0401** | 35 (56.4 %) | 12 (44.4 %) | *1.000* |
| **Phl p 1** | 36 (58.1 %) | 13 (48.1 %) | *1.000* |
| **Lol p 1** | 36 (58.1 %) | 13 (48.1 %) | *1.000* |
| **Secc pollen** | 19 (30.6 %) | 9 (33.3 %) | *0.4629* |
| **Can f 1** | 23 37.1 %) | 13 (48.1 %) | *0.4888* |
| **Der f 2** | 35 (56.4 %) | 13 (48.1 %) | *1.000* |
| **Der p 2** | 36 (58.1 %) | 11 (40.7 %) | *0.7254* |
| **Der p 23** | 27 (43.5 %) | 11 (40.7 %) | *0.7337* |
| **Fel d 1** | 26 (41.9 %) | 15 (55.5 %) | *0.2911* |
| **Alt a 1** | 14 (22.6 %) | 7 (25.9 %) | *0.4453* |
| **Gal d white** | 12 (19.3 %) | 10 (37.0 %) | *0.1280* |
| **Gal d yolk** | 6 (9.7 %) | 5 (18.5 %) | *0.5977* |
| **Mala s 11** | 14 (22.6 %) | 12 (44.4 %) | *0.0693* |

**Explanation**

Aln g 1 (PR-10 protein, alder), Bet v 1 (PR-10 protein, birch), Cor a 1.0401 (PR-10 protein, hazel pollen), Phl p 1 (Beta expansin, Timothy grass), Lol p 1 (Beta expansin, rye grass), Sec c_pollen (cultivated rye, pollen), Can f 1 (Lipocalin, dog), Der p 2 (NPC2 family, house dust mite), Der f 2 (NPC2 family, house dust mite), Der p 23 (peritrophin-like protein domain, house dust mite), Fel d 1 (uteroglobin, cat), Alt a 1 (unknown, Alternaria alternata), Gal d white – egg, Gal d yolk – egg, Mala s 11 (Mn superoxide dismutase, Malassezia sympodialis)

**Complement to Table 3.** The statistical comparison of the levels of interleukins between patients with and without dupilumabl therapy compared to control group.

|  | **Dupilumab yes**  **27 patients** | | **Dupilumab no**  **62 patients** | | **Control group**  **44 patients** | | **KW test**  ***p - value*** | **DUP yes**  **/DUP no** | **DUP no /control** | **DUP yes**  **/ control** |
| --- | --- | --- | --- | --- | --- | --- | --- | --- | --- | --- |
|  | mean | St. deviation | mean | St. deviation | mean | St. deviation |  |  |  |  |
| IL 4 uns | 0.0437 | 0.132 | 0.0137 | 0.0669 | 0.0157 | 0.0601 | 0.854 |  |  |  |
| IL 4 s | 0.0851 | 0.136 | 0.0304 | 0.0803 | 0.0310 | 0.0703 | 0.0584 | **<0.05** |  |  |
| IL 13 uns | 0.0544 | 0.192 | 0.0130 | 0.0839 | 0.0227 | 0.115 | 0.294 |  |  |  |
| IL 13 s | 0.128 | 0.323 | 0.0971 | 0.281 | 0.0348 | 0.138 | 0.265 |  |  |  |
| IL 5 s | 0.0765 | 0.106 | 0.0512 | 0.0834 | 0.0281 | 0.0748 | 0.0245 |  |  | **<0.05** |
| IL 10 s | 0.477 | 0.447 | 0.319 | 0.359 | 0.265 | 0.510 | 0.0009 |  | **<0.05** | **<0.001** |
| IL 33 uns | 0.107 | 0.396 | 0.0252 | 0.177 | 0 | 0 | 0.203 |  |  |  |
| IL 33 s | 0.100 | 0.376 | 0.0230 | 0.167 | 0 | 0 | 0.203 |  |  |  |

We show the mean and standard deviation (s.d.) of interleukins (in picogram per millilitre). The levels of IL5 unstimulated and IL10 unstimulated is zero in all groups, so comparison could not be made. The hypothesis of agreement was tested against the alternative that at least two groups differ from each other. Nonparametric Kruskal-Wallis analysis of variance with post-hoc Dunn's test with Bonferroni modification of significance level was used. The respective significance levels are highlighted.

We show a statistically significant difference when comparing the groups:

1) AD patients with dupilumab versus AD patients without dupilumab (DUP yes/DUP no).

2) AD patients without dupilumab versus control (DUP no/control)

3) AD patients with dupilumab versus control (DUP yes/control

Explanation: uns. - unstimulated interleukins, s.- stimulated interleukins, KW test = Kruskal-Wallis analysis. DUP: dupilumab.

**Complement material to section 2.2.**

According to the strictly followed instructions for the Luminex test, the standard samples were prepared using three-fold dilutions. The standard curve therefore has a rather exponential character. Example of a standard curve for IL-4 is recorded in Complement materiál to section 2.2.


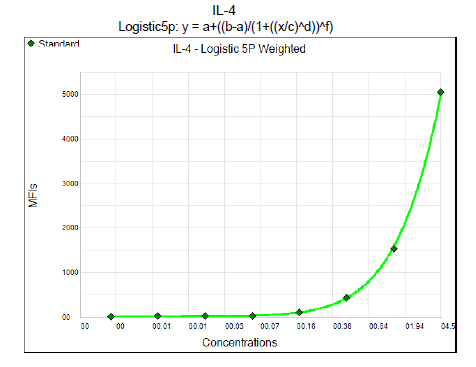


We also attach an example of specifically stated concentration values:


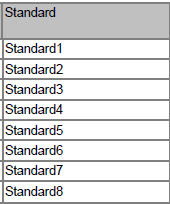

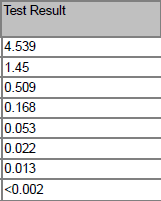

Supplement: Supplementary Table 2 — The statistical comparison of the levels of interleukins between patients with and without dupilumabl therapy compared to control group. We show the mean and standard deviation (s.d.) of interleukins (in picogram per milliliter). The levels of IL5 unstimulated and IL10 unstimulated is zero in all groups, so comparison could not be made. The hypothesis of agreement was tested against the alternative that at least two groups differ from each other. Nonparametric Kruskal-Wallis analysis of variance with post-hoc Dunn’s test with Bonferroni modification of significance level was used. The respective significance levels are highlighted. We show a statistically significant difference when comparing the groups: 1) AD patients with dupilumab versus AD patients without dupilumab (DUP yes/DUP no). 2) AD patients without dupilumab versus control (DUP no/control). 3) AD patients with dupilumab versus control (DUP yes/control. Explanation: uns. - unstimulated interleukins, s.- stimulated interleukins, KW test = Kruskal-Wallis analysis. DUP: dupilumab. [file Table1.docx]
